# Germination patterns and seedling growth of congeneric native and invasive *Mimosa* species: Implications for risk assessment

**DOI:** 10.1002/ece3.11312

**Published:** 2024-04-22

**Authors:** Nisha Kharel, Anuj Dangol, Ashmita Shrestha, Hemanti Airi, Anjana Devkota, Lal Bahadur Thapa, Bharat Babu Shrestha

**Affiliations:** ^1^ Central Department of Botany Tribhuvan University Kathmandu, Kirtipur Nepal

**Keywords:** biomass allocation, functional traits, *Mimosa diplotricha*, *M. himalayana*, relative growth rate, species invasiveness, Timson's index

## Abstract

Comparisons of plant traits between native and invasive congeners are useful approaches for identifying characteristics that promote invasiveness. We compared germination patterns and seedling growth of locally sympatric populations of native *Mimosa himalayana* and two varieties of invasive *M. diplotricha* (var. *diplotricha* and var. *inermis*) growing in southeastern Nepal. Seeds were germinated under a 12‐h photoperiod or complete dark, low (25/15°C day/night) and high (30/20°C) temperatures, different water stress levels (0, −0.1, −0.25, −0.5, −0.75 and −1.0 MPa), and soil depths (0, 2, and 4 cm). Plant height, biomass allocations, and relative growth rate (RGR) of seedlings were measured. Invasive *M. diplotricha* had higher germination percentage, rate, and shorter germination time compared with the native species. Germination of both congeners declined as water stress increased, but the decline was more pronounced in native species. Seedling emergence declined with increasing depth in all taxa. The seedlings of invasive species were taller with higher leaf number and allocated greater proportion of biomass to shoot, whereas the native congener allocated greater biomass to root. The RGR was nearly twice as high in invasive species as it was in the native congener. Seedling height and number of leaves were always higher in invasive than in native species, and the native–invasive differences increased over time. Better germination and higher growth performance of invasive species than the congeneric native one suggests that seed germination and seedling growth can be useful traits for the prediction of species' invasiveness in their introduced range during risk assessment process.

## Graphical abstract

Comparing the traits of congeneric native and invasive plants provides insights on the invasiveness of the introduced species. We compared germination pattern and seedling growth vigor of native *Mimosa himalayana* and invasive *M. diplotricha* which are sympatrically occurring in southeastern Nepal. The invasive species had better germination (higher germination rate) and higher seedling growth vigor (higher relative growth rate) than the congeneric native species, suggesting that seed germination and seedling growth traits can be useful in weed risk assessment to identify potentially invasive species.

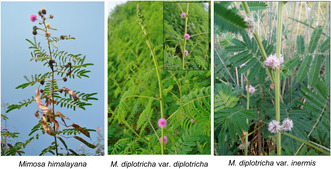

## 1 INTRODUCTION

Understanding what causes some species to become invasive has received a lot of attention because only a small percentage of introduced species become invasive (Williamson & Fitter, 1996). Identification of the factors that contribute to successful invasion is essential for predicting the emergence and expansion of invasive species and formulating measures to their prevention (e.g., through weed risk assessment) and control (Groves et al., 2003; Kolar & Lodge, 2001; Koop, 2012). In general, plant functional traits, such as the ones related to physiology, biomass allocation, growth rate, size, and fitness may determine invasiveness (Grotkopp et al., 2002; Kolar & Lodge, 2001). These functional traits can also be used to predict invasive species' performances in ecosystems (Kaushik et al., 2022) and some of these traits (e.g., seed size, capacity to propagate through vegetative parts) have therefore been routinely used in weed risk assessments (Groves et al., 2003). The performance of invasive species (i.e., invasiveness) also depends on the relative position of co‐occurring native and invasive species in the functional trait space (Hui et al., 2021). Additionally, the use of plant functional traits as input variables in ecological niche modeling may improve the accuracy of model predictions (Wang & Wan, 2021). Several studies have revealed plant characteristics that frequently correspond with invasion success. These include size (height/biomass), growth rate, and competitiveness (van Kleunen et al., 2010). However, there are very few generalizations that can be made on the role of plant functional traits for invasiveness (i.e., a capacity of introduced species to establish, spread and inflict impacts on native species, ecosystem and environment in a reasonably short period) among alien species and across studies (Hayes & Barry, 2008), despite massive increase in literature of invasion ecology (Pyšek et al., 2008). It is because possession of a set of plant traits per se may not enable a plant to be invasive as the species' invasiveness also depends on a range of other biotic (e.g., presence of herbivores, pathogens, native competitors, traits of the recipient community) and abiotic (e.g., climatic suitability, nutrient, and other resources availability) factors which vary across habitats and geographic regions (Catford et al., 2019; Chun et al., 2010; Hui et al., 2023; Koop, 2012). Therefore, it can be challenging to pinpoint the traits that are consistently linked to invasiveness (Alpert et al., 2000), yet identification of such potential traits is an essential prerequisite for explaining and predicting invasions (Rejmánek, 1996). Research interests on these topics have increased in recent years (e.g., Catford et al., 2019; Liu et al., 2022; Milanović et al., 2020), yet the available data remain inadequate to draw any generalization globally and across taxonomic groups. Further, understanding the differences between co‐occurring native and invasive species in key functional traits has been identified as one of the major research gaps to comprehend species' performance in dynamic ecosystems (Kaushik et al., 2022). It warrants additional studies with taxa and site‐specific analyses which can provide better insights (Hayes & Barry, 2008). However, the majority of studies of species' traits associated with invasiveness have been limited to relatively simple traits, such as plant height and growth form that are easily available (van Kleunen et al., 2010). Other traits such as those related to seed germination are considered important to predict invasiveness (Perglová et al., 2009), but some studies have reported no significant differences in terms of germination between successful and unsuccessful invaders (Radford & Cousens, 2000; Reichard, 2003). Therefore, additional studies comparing native–invasive congeners under a common environment is needed to further characterize and identify any potential differences in seed germination and seedling growth traits, which can subsequently provide valuable insights on species' invasiveness (Chauhan & Johnson, 2008; Graebner et al., 2012; Schlaepfer et al., 2010; van Kleunen et al., 2010). Furthermore, comparisons between congeneric native and invasive species sharing the same habitat provide additional opportunities to assess whether the invasive species can displace a native species (Munoz & Ackerman, 2011; Webb et al., 2002). Therefore, a comparison of germination patterns and seedling growth vigor between congeneric native and invasive species can be an effective approach to identify traits useful for the prediction of invasiveness.

Seed germination rate and timing of invasive species may have significant impacts on coexisting native species, as early germination of invasive species may be an advantageous event in survival, growth, and subsequent establishment in competitive environments due to space preemption and greater access to resources (Byun, 2023; Guido et al., 2017). Invasive species tended to have higher germination percentage (GP) and shorter time to germination than the co‐occurring or congeneric native species (Cervera & Parra‐Tabla, 2009; Munoz & Ackerman, 2011; Wainwright & Cleland, 2013). For example, invasive *Ardisia elliptica* (Myrsinaceae) has higher GP and shorter mean germination time (MGT) than the sympatric native *A. obovata* in Puerto Rico (Munoz & Ackerman, 2011). Tolerance of invasive *Ruellia nudiflora* (Acanthaceae) to temperature and water stress extremes during germination is higher than that of native *R. pereducta* in Mexico (Cervera & Parra‐Tabla, 2009). After germination, the invasive plants tend to grow more quickly than native species (Grotkopp & Rejmánek, 2007), or they have traits associated with higher relative growth rates (RGR) compared with native species (Hamilton et al., 2005). For example, James and Drenovsky (2007) reported a higher RGR of invasive species (6 spp.) than co‐occurring native species (6 spp.) in Oregon, USA. Similarly, invasive species *Prosopis juliflora* and *Leucaena leucocephala* were reported to use light and nutrients more efficiently compared with native species, *Mimosa caesalpiniifolia* and *Myracrodruon urundeuva*, favoring growth and resources acquisition even under the availability of limited water resources (Barros et al., 2020). However, Munoz and Ackerman (2011) reported a lack of difference in the growth attributes such as the RGR between invasive *Ardisia elliptica* and native *A. obovata*. A meta‐analysis also revealed that the difference between invasive and native in terms of RGR is not always significant and highly dependent on growing conditions (Daehler, 2003).

As summarized above, the empirical evidences are either inadequate (germination pattern) or inconsistent (growth rate) to draw any generalization regarding the distinction between invasive and native plant species. This necessitates additional studies particularly in data‐poor regions such as South Asia. In this context, we studied the seed germination patterns and seedling growth of sympatric populations of native *Mimosa himalayana* and two varieties of recently reported, invasive *M. diplotricha* in southeastern Nepal. The native *M. himalayana* has no record of introduced population outside of its native distribution range in South and Southeast Asia (POWO, 2023). Invasive *M. diplotricha* has been reported to be a major threat to forest ecosystems, agricultural land, and pastures in tropical to subtropical Asia and the Pacific regions (Sankaran & Suresh, 2013). In Nepal, the species has been reported to invade only in southeastern Nepal (Sharma et al., 2020), where massive environmental (e.g., smothering native plant species) and economic impacts (e.g. livestock death as a result of poisoning) of the species have been already observed (personal observations of BBS and NK during multiple visits to southeastern Nepal in 2021 and 2022). Additionally, it also shares habitat with native congener *M. himalayana* in southeastern Nepal. We hypothesize that the invasive *M. diplotricha* outperforms the sympatric and native congener *M. himalayana* (which has no introduced population elsewhere) in seed germination and seedling growth. To test this hypothesis, we put forth the following questions: (1) Do invasive *M. diplotricha* perform better in germination than the native *M. himalayana*? (2) Is extreme water stress tolerance of invasive higher than that of native during germination? and (3) Is seedling growth performance of invasive better than that of native? Results of this study provide useful insights into the invasiveness of *Mimosa* species and can be helpful in detecting potentially invasive species during risk assessment.

## 2 MATERIALS AND METHODS

### 2.1 Study species

**FIGURE 1:**
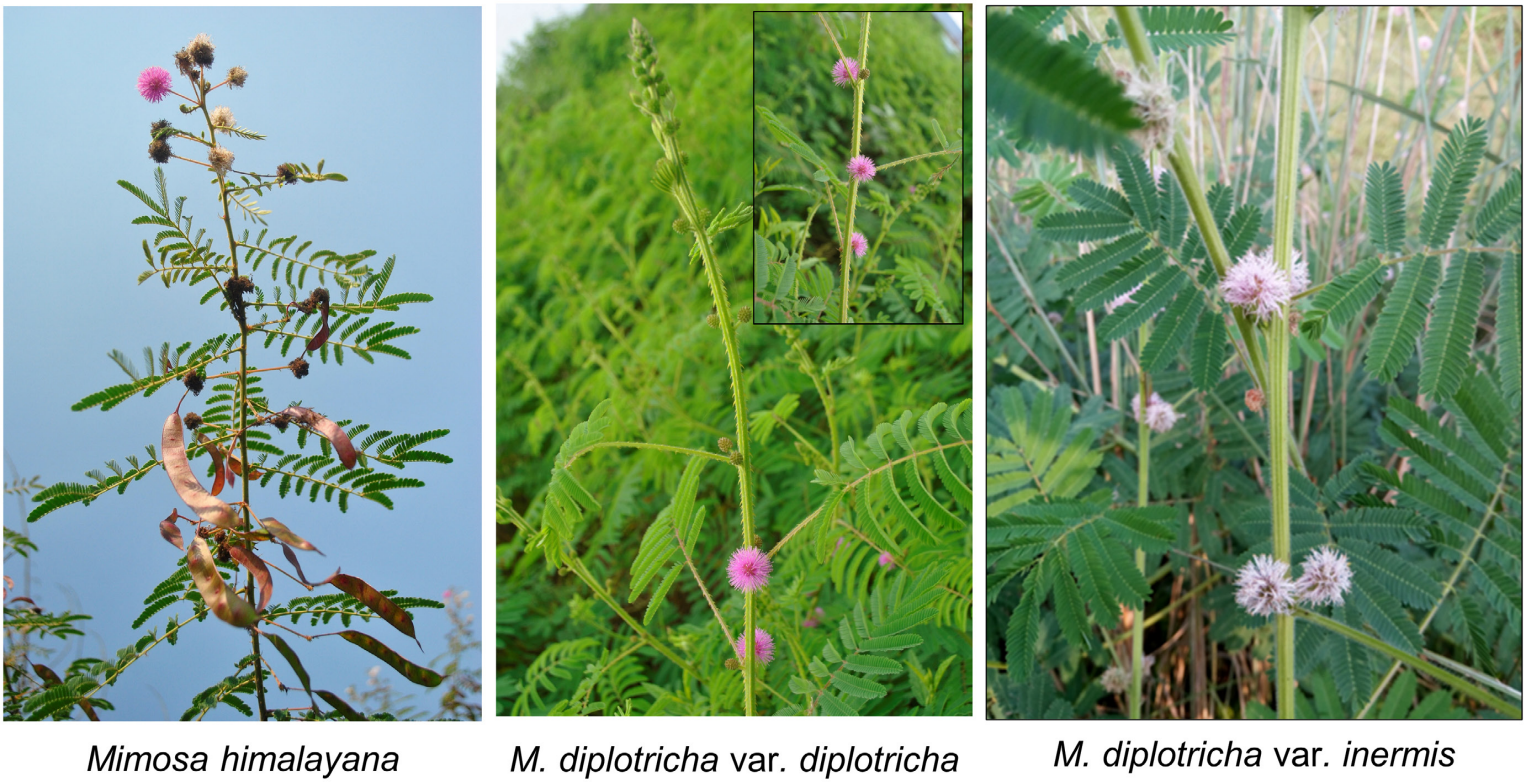
Flowering twig of *Mimosa* species from the seed collection sites in southeastern Nepal.

Sympatric populations of native *Mimosa himalayana* Gamble and invasive *M. diplotricha* Wright ex Sauvalle (Fabaceae) from the Jhapa district located at southeastern Nepal were selected for the present study. *Mimosa himalayana* is a scrambling shrub native to Himalaya (Grierson & Long, 2001; Shrestha et al., 2022), which is not known outside of its native distribution range (POWO, 2023). *Mimosa diplotricha* is a subshrub native to tropical Central and South America, which is invasive in more than 45 countries in Asia, Africa, and Oceania including Nepal (Sharma et al., 2020; Uyi, 2020). It appears that the weed was introduced intentionally outside of its' native range for soil bioengineering, as cover crop, as green manure, or as hedge plant. The weed produces numerous small seeds (10,000 seeds per plant per annum) which are dispersed by animals (spiny pod clusters being adhered to animal fur), water, and as contaminants of agriculture produces and construction materials (e.g., sand, gravel) (Parsons & Cuthbertson, 1992). *Mimosa diplotricha* is known to have significant negative impacts on livelihood through reduced forage production in range lands, interfering human movements, reduced abundance of medicinal plants, and crop yield loss (Witt et al., 2020). Consumption of this weed by cattle may lead to their death because of nephrotoxicity (Sankaran & Suresh, 2013; Shridhar & Kumar, 2014). Two varieties of *M. diplotricha* have been reported in Nepal (Sharma et al., 2020). *Mimosa diplotricha* var. *diplotricha* Sauvalle has prickles on the stem surface while *M. diplotricha* var. *inermis* (Adelbert) Veldkamp is without prickles (Wu et al., 2010) (Table 1, Figure 1). The occurrence frequency of *M. diplotricha* var. *diplotricha* in southeastern Nepal and its invasiveness (based on the extent of area invaded) are higher than that of *M. diplotricha* var. *inermis* (Sharma et al., 2020; personal observations of BBS).

**TABLE 1:** Taxonomic and biogeographic account of the study species.

Attributes	Mimosa himalayana Gamble	*Mimosa diplotricha* C. Wright ex Sauvalle
Synonym	*Mimosa rubicaulis* subsp. *himalayana* (Gamble) H. Ohashi	*Mimosa invisa* Mart.
Common name	Himalayan Mimosa	Giant sensitive plant
Local vernacular name	*Areli* [Nepali]	*Aara kande, Ulta kande, Thulo Lajjawati Jhar* [Nepali]
Habit	Scrambling shrub of 4–5 m	Subshrubs with scandent or prostrate stems up to to 5 m
Morphological description	Leaves with 16–20 pairs of leaflets which in turn have 6–9 pairs of pinnae (2.5–6 cm long); bears pink flower heads of approximately 1.5 cm diameter on axillary cluster of 3–4 on peduncles (Grierson & Long, 2001).	Stem is quadrangular with or without prickles along angles; leaves measure 10–15 cm with 3–7 or 10 pairs pinnae, each pinna measuring 2–4.5 cm consisting of 11–20 or 30 pairs of leaflets; 1 or 2 axillary flower heads of ca. 1 cm diameter on 5–10 mm peduncles; seed pods in clusters and seeds are yellow‐brown (Wu et al., 2010)
Flowering and fruiting phenology	Flowering: June–August Fruiting: August–January	Flowering: October–November Fruiting: November–January
Native distribution	Himalaya, Assam, Burma and South Asia	Tropical South America, the Caribbean, and Central America
Introduced range	Not known outside native range	>45 countries in Asia, Africa, and Oceania (Uyi, 2020)
Distribution in Nepal	Between 100 and 1900 m above sea level in eastern, central, and western Nepal (Shrestha et al., 2022)	Sunsari, Morang and Jhapa districts in southeastern Nepal
First report in Nepal	—	2019 (Sharma et al., 2020)

### 2.2 Seed collection

**FIGURE A1:**
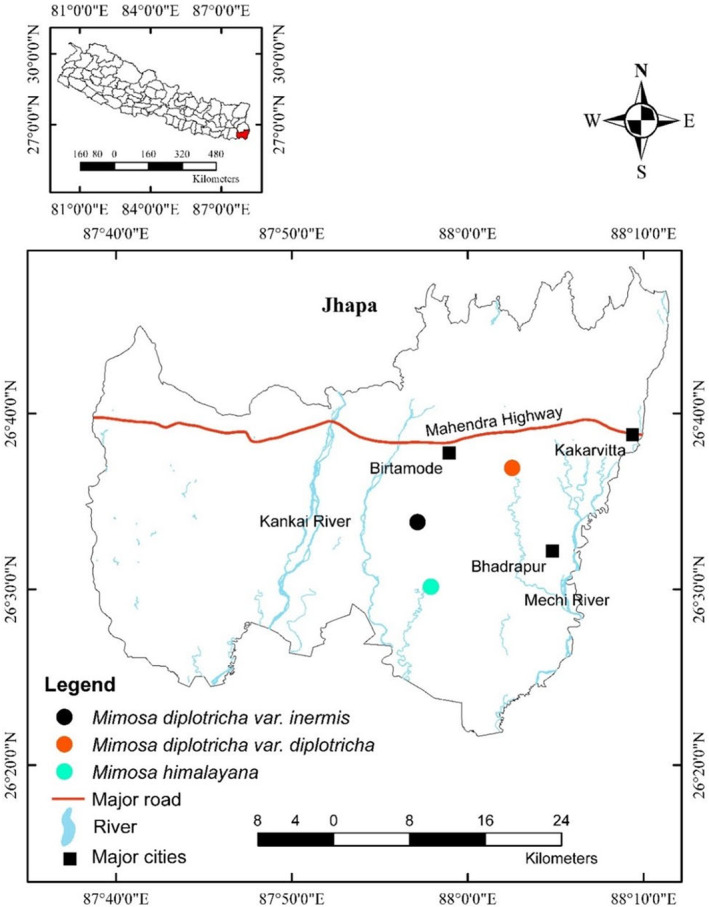
Seed collection sites of *Mimosa* species in Jhapa, eastern Nepal.

Ripe seeds of each taxon were collected from ca. 10 × 10 m^2^ plot from Jhapa district in southeastern Nepal (Table 2, Figure A1). The region has dry tropical climate with hot and humid summer with monsoon rain (June through September) and cold and dry winter. The collected seeds were cleaned upon returning to the laboratory, placed in air‐tight plastic containers, and stored in the refrigerator (4°C) for about 5 days until they were used in germination experiments.

**TABLE 2:** Details of seed collection sites.

Collection details	*Mimosa himalayana*	*M. diplotricha* var. *diplotricha*	*M. diplotricha* var. *inermis*
Collection date	January 7, 2022	January 8, 2022	January 9, 2022
Coordinates	26.5024° N, 87.9651° E	26.6150° N, 88.0421° E	26.5636° N, 87.9524° E
Elevation (m)	85	106	102
Collection site	Haldibari	Birtamode	Barhadashi
Vegetation of the collection sites	Forest edge	Forest understory	Roadside vegetation

### 2.3 Seed size and mass measurements

**FIGURE A2:**
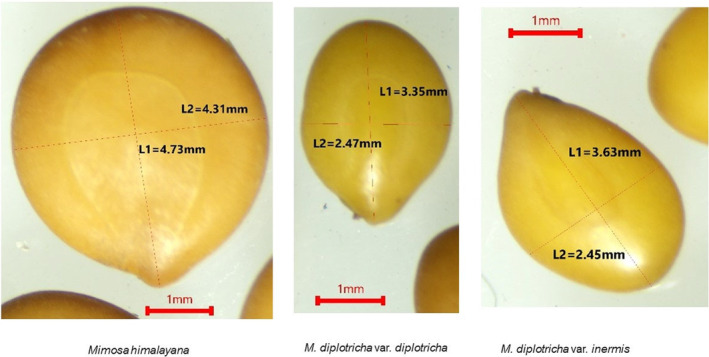
Seed size measurement of *Mimosa* species.

Seed mass was measured in three lots of 50 healthy seeds each after oven drying for 48 h at 70°C using digital balance (0.0001 g) (Baskin & Baskin, 2014). Seed size was measured by trilocular stereomicroscope using “Wave Image” software. Twenty seeds of each taxon were selected randomly and their length (longest axis length of the seed starting from hilum) and breadth (second longest axis perpendicular to the length axis) were measured (Figure A2).

### 2.4 Test for imbibition of water

**FIGURE A3:**
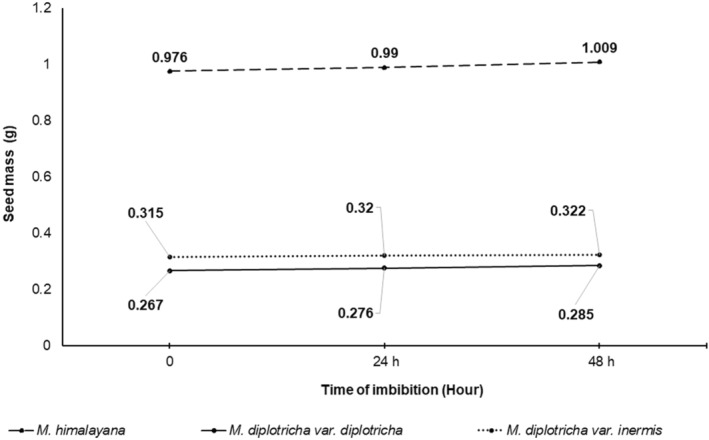
Change in seed mass (per 50 seeds) after imbibition for 24 and 48 h.

To determine if the seeds were permeable to water, we measured the dry mass of 50 seeds per taxon and transferred them to a Petri dish containing water. These seeds were re‐weighed after 24 and 48 h. In each measurement, seeds were removed from the Petri dish, blotted dry, and weighed using digital balance (Baskin & Baskin, 2014). Seed mass of all taxa did not increase substantially even 48 h after placing them in water (Figure A3), suggesting that seed coat was impermeable and the seeds had physical dormancy (Baskin & Baskin, 2014). Physical dormancy of *Mimosa* was further confirmed by a rapid germination of seeds after rupturing seed coats in a preliminary experiment (data not presented here).

### 2.5 Seed scarification

Seed scarification was required to break physical dormancy of *Mimosa* species. Following the suggestion of Chauhan and Johnson (2009), hot water scarification was used because it is more convenient and environment‐friendly than other methods of scarification such as the mechanical methods and use of acids. For hot water scarification, seeds were wrapped in muslin cloth as a loose pouch and dipped in 70°C hot water for 10 minutes (Chauhan & Johnson, 2009). Soon after scarification, the pouches were removed from hot water and opened immediately to bring seeds to room temperature, and subsequently they were transferred to Petri dishes for germination experiments.

### 2.6 Germination experiments

#### 2.6.1 Germination under different environmental conditions

**FIGURE A4:**
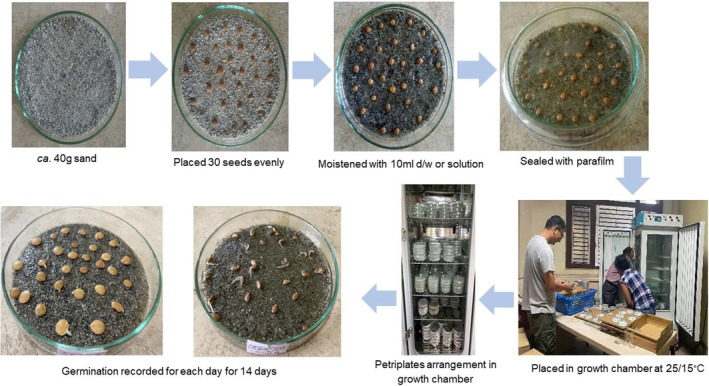
Different stages of seed germination experiment.

Scarified seeds were germinated under different environmental conditions, such as light and dark, low and high temperatures, and different levels of water stress. Thirty seeds of each taxon were placed in 9‐cm diameter Petri dish, and there were five replicates for each treatment. Thus, 150 seeds were tested altogether in each treatment. Sand was used as a substrate which was moistened with either distilled water or polyethylene glycol 6000 (PEG) solution. The Petri dishes with seeds were sealed by parafilm tape and placed in a growth chamber (Model: GC‐300TLH, Jeio Tech, Korea) (Figure A4). Light intensity (fluorescent light) inside the chamber was ca. 3000 lux and relative humidity was 70%. Seeds were further maintained under the following conditions:

- Light and dark: Seeds were allowed to germinate under 12 h photoperiod (12/12 h, light/dark), and continuous dark was maintained by wrapping the Petri dishes with double layer aluminum foil.
- Low and high temperatures: Seeds were allowed to germinate at low (25/15°C, day/night) and high (30/20°C) temperatures. At both temperatures, seeds were exposed to either a 12‐h photoperiod or continuous dark as mentioned above.
- Water stress: Water stress was induced by germinating seeds in PEG solutions of different water potentials (−0.1, −0.25, −0.5, −0.75, and −1 MPa). Stock PEG solution (−1 MPa) was prepared by dissolving 296 g of PEG in 1 L distilled water, which was further diluted to prepare other solutions of different water potential (Michel & Kaufmann, 1973). Distilled water (0 MPa) was used as a control. Capacity of the study species to germinate under water stress condition provides valuable information on their reproductive phenology because the seed dispersal of both species occurred at the onset of the dry season which lasts for 5–8 months (before monsoon begins in June).

The number of germinated seeds in each Petri dish was counted daily for 14 days. At each count, the germinated seeds were removed to reduce the crowding of seedlings. Emergence of radicle was used as a criterion for germination of seeds (Baskin & Baskin, 2014). However, the germination of seeds maintained at dark was recorded at the end of experiment.

#### 2.6.2 Seedling emergence

To evaluate the impact of seed sowing depth on seedling emergence, seeds were sown at a predefined depth in pots (height 13 cm, diameter 11 cm) filled with prepared substrate (sand, vermicompost, and cocopeat in the ratio of 7:2:1 by volume). The pots were enamel painted with a black color to block out all light except from the upper surface. In each pot, 30 scarified seeds were sown at various depths: 0 (surface), 2, 4, 6, 8, and 10 cm. Seeds were sown, covered with the substrate at the marked depth, and watered slowly to field capacity. Further watering (10 mL to each pot) was done at 1 day interval. The number of seedlings that emerged above the soil surface was recorded daily. Seeds were considered to emerge when the cotyledonary leaves spread out completely above the substrate surface. Emerged seedlings were removed daily and the experiment was terminated 30 days after seed sowing.

### 2.7 Raising seedlings and biomass harvest

**FIGURE A5:**
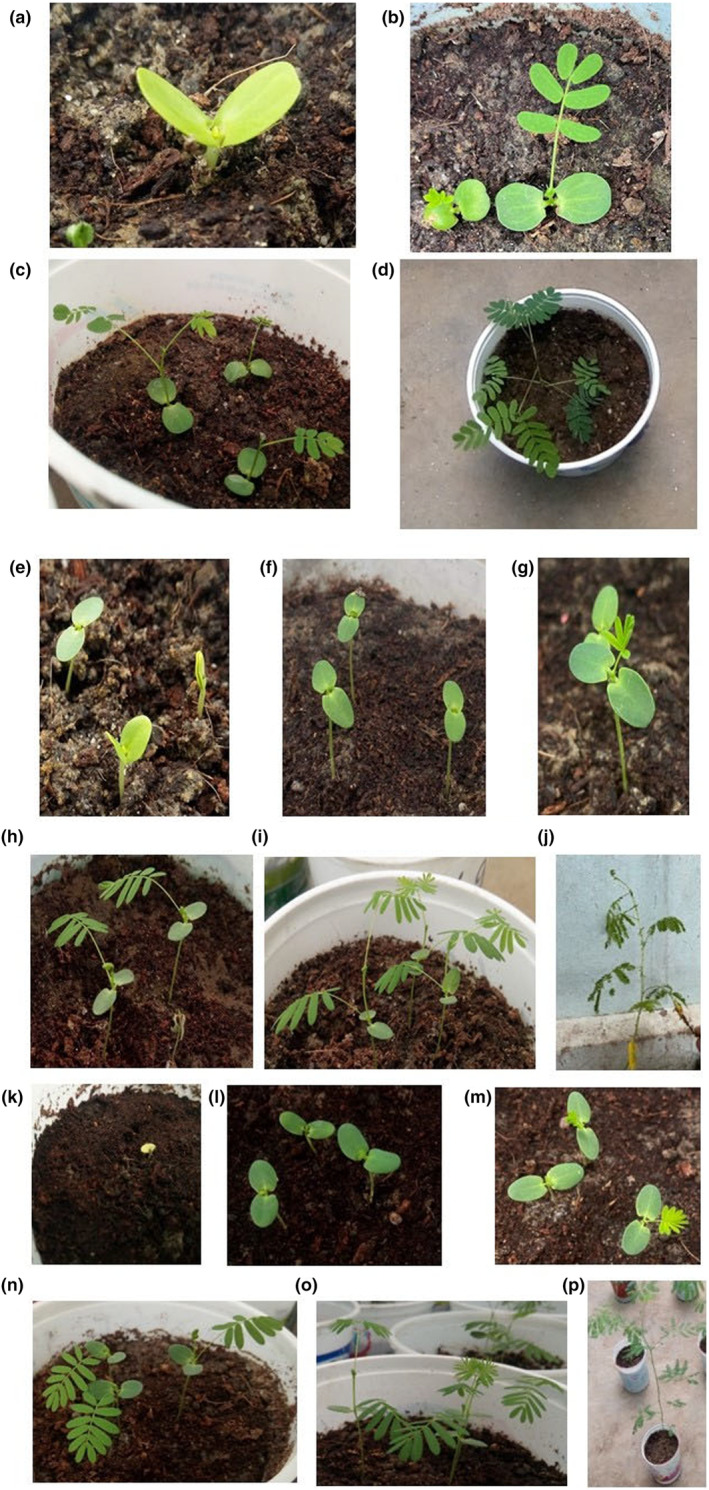
Different stages of seedling growth of *Mimosa himalayana* (a–d), *M. diplotricha* var. *diplotricha* (e–j) and *M. diplotricha* var. *inermis* (k–p).

Some functional traits, such as height, biomass allocation, and RGR were measured in seedlings grown in a greenhouse (Figure A5). For raising seedlings, plastic pots (height 13 cm, diameter 11) were filled with a mixture of soil, sand, vermicompost, and cocopeat prepared in the proportions of 3:3:3:1 by volume. The substrate was saturated to field capacity by tap water (ca. 250 mL). Then, three scarified seeds were sown in each pot, with 80 pots for each taxon. The pots were maintained in a greenhouse with a mean temperature of 27 ± 3°C and the light intensity ca. 6220 lux measured at 11–12 a.m. On every alternative day, the pots were rearranged to lessen the positional effects. Watering (10 mL per pot) was done daily. Seeds of both varieties of *Mimosa diplotricha* began to germinate 5 days after sowing (DAS), whereas the seeds of *M. himalayana* began to germinate at 10 DAS. Each pot had two to three seedlings depending on the number of seeds germinated. Two seedlings were maintained in each pot after 20 DAS from the pot where there were three seedlings. Finally, only one seedling was maintained in each pot after 38 DAS by removing seemingly less vigor seedling. While doing so, we tried to ensure for each taxon that all pots had seedlings that were nearly of the same height and were healthy. Some of the seedlings died and the seedling mortality of native *Mimosa himalayana* was higher (20%) than the invasive congener (5% in *M. diplotricha* var. *diplotricha* and 6% in *M. diplotricha* var. *inermis*).

The first biomass harvest of seedling was done on 48 DAS. Twenty seedlings of each taxon were drawn at random by a lottery method. The number of leaves of each seedling were counted and subsequently excised along with petiole to determine leaf biomass. Then, the seedlings were removed from the pots along with soil and gently washed to remove all soil particles from the root. Shoot and root lengths of each seedling were measured and they were separated to determine the respective biomass. Fresh root, stem, and leaf parts of each seedling were packed separately in paper envelope and were oven dried at 60°C for 72 h to determine the dry biomass (digital balance, 0.0001 g). Subsequent three harvests were made in the same way at an interval of 14 days (i.e., 62, 76, and 90 DAS). Due to seedling mortality, the number of seedlings available for each harvest ranged from 12 to 20 for each taxon (Table A1).

### 2.8 Data analyses

Daily records of seed germination in Petri dishes were used to calculate germination percent (GP), Timson's index (TI), and MGT following the methods suggested by Baskin and Baskin (2014). The GP was the number of seeds germinated as the percentage of the total number of seeds sowed. The TI, a measure of germination rate, was calculated as the sum of cumulative daily GP obtained for each Petri dish. Since the germination experiment was continued to 14 days, the maximum possible value of TI was 1400. Finally, the MGT, a measure of time it takes for most of the seeds to germinate, was calculated as $\sum_{i=1}^{k}\begin{array}{c} \;\\ niti/\sum_{i=1}^{k}ni \end{array}$, where t_*i*_ was the time from the start of experiment to the *i*th observation (day), n_*i*_ was the number of seeds germinated at time *i*, and *k* was the last day of germination experiment (Baskin & Baskin, 2014). To assess the impacts of seed sowing depth on seedling emergence, the number of emerged seedlings was expressed as the percentage of total seeds sown.

Biomass allocation was analyzed by expressing the biomass of root, stem, and leaf as a fraction of total seedling biomass (Poorter et al., 2012). For example, the root mass fraction (RMF) was obtained as the ratio of root biomass of a seedling and total biomass of the same seedling. In the same way, stem mass fraction (SMF) and leaf mass fraction (LMF) were calculated. Similarly, root to shoot ratio (RS) was obtained after root biomass was divided by shoot biomass (sum of leaf and stem biomass).

**FIGURE A6:**
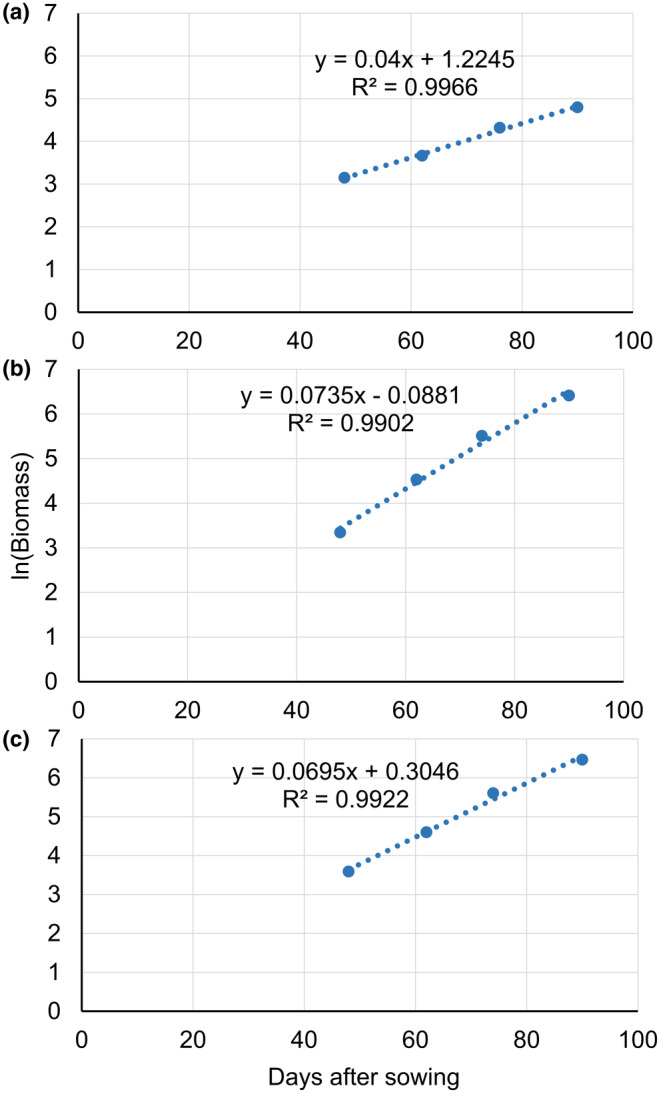
Variation of seedling biomass (ln transformed) with days after sowing of (a) *Mimosa himalayana* (b) *M*. *diplotricha* var. *diplotricha* (c) *M. diplotricha* var. *inermis*. Slope of the fitted line obtained from the regression equation was considered as the relative growth rate (RGR).

RGR was determined as slope of linear regression line obtained by plotting seedling biomass against days after seed sowing (DAS) of each harvest (Perez‐Harguindeguy et al., 2013). Total biomass of each seedling was log transformed (ln biomass) and the mean value of ln biomass was calculated for each taxon and each harvest. Then, mean ln biomass was plotted against DAS to get linear regression equation (Figure A6).

Prior to statistical analyses, seed GP was converted to fraction and then square root and degrees arcsine transformed, while fraction values of biomass allocations (RMF, SMF, LMF and RS) of each seedling were square root and arcsine transformed to improve homoscedasticity (Baskin & Baskin, 2014; Sokal & Rohlf, 1995). Other values, such as Timson's index, MGT, seedling height, and the number of leaves of seedlings met the assumption of homoscedasticity (Levene's test). We performed one‐way analysis of variance (ANOVA) to analyze the seed size among three taxa with a Tukey's post hoc test. An independent sample *t*‐test was used to compare the mean of germination parameters of each species between a 12‐h photoperiod and dark as well as low and high temperature conditions. One‐way ANOVA with Tukey's post hoc tests were also used to compare the mean of germination parameters under different environmental conditions (light, temperature, and water stress) among taxa. To analyze the effect of water potential (or seed sowing depth in case of seedling emergence experiment), species, and their interactions, two‐way ANOVA was done. To analyze the effect of depth of seed sowing on emergence within and among taxa, we performed one‐way ANOVA using a Tukey's post hoc test. One‐way ANOVA was also used to analyze the differences in biomass allocation patterns (RMF, SMF, LMF, and RS), plant height, and leaf number between species at each harvest and within species at different harvests. Statistical Package for Social Science (SPSS, ver. 25) was used for all statistical analyses (IBM Corp., 2017).

## 3 RESULTS

### 3.1 Seed mass and seed size

Native *Mimosa himalayana* had greater seed mass and larger seed size (length and breadth) compared with invasive congener *M. diplotricha* var. *diplotricha* as well as *M. diplotricha* var. *inermis* (Table 3). The seed mass of native species was three times greater than that of invasive species.

**TABLE 3:** Seed mass and size of *Mimosa* species (mean ± SD).

Taxa	Seed mass (mg)	Seed size	
		Length (mm)	Breadth (mm)
*Mimosa himalyana*	18.9 ± 1.4	4.3 ± 0.3^b^	3.9 ± 0.4^b^
*Mimosa diplotricha* var. *diplotricha*	5.5 ± 0.2	3 ± 0.3^a^	2.2 ± 0.3^a^
*Mimosa diplotricha* var. *inermis*	6 ± 0.2	3.2 ± 0.2^a^	2.3 ± 0.1^a^

*Note*: Different alphabets (a, b) in superscript across each column represent a significant difference between mean (ANOVA, *p* ≤ .05). The ANOVA was not run for seed mass due to only three replicate samples.

### 3.2 Effects of light and temperature

There was no significant difference (*p* > .05) in seed GP between high (30/20°C) and low (25/15°C) temperatures as well as a 12‐h photoperiod and complete dark for each taxon (Table 4, statistical results not shown). However, across taxa, the native taxon had lower germination rate at both high and low temperatures than the invasive taxon in photoperiod condition. In contrast, there was no difference in GP among three taxa at high temperature in dark. However, the germination of *M. diplotricha* var. *inermis* was higher compared with *M. himalayana* and *M. diplotricha* var. *diplotricha* at low temperature in dark (Table 4).

**TABLE 4:** Effect of light and temperature on germination percentage (mean ± SE).

Taxa	Photoperiod (12 h)		Dark	
	High temperature	Low temperature	High temperature	Low temperature
*Mimosa himalayana*	91 ± 2^a^	95 ± 2^a^	96 ± 2^a^	98 ± 1^a^
*Mimosa diplotricha* var. *diplotricha*	97 ± 1^b^	96 ± 1^a^	97 ± 1^a^	98 ± 1^a^
*Mimosa diplotricha* var. *inermis*	99 ± 1^b^	100 ± 0^b^	99 ± 1^a^	100 ± 0^b^

*Note*: Different alphabets (a, b) in superscript across each column represent a significant difference between mean (ANOVA, *p* ≤ .05); there was no significant difference between high and low temperatures as well as photoperiod and dark conditions within each taxon (independent sample *t*‐test).

**FIGURE 2:**
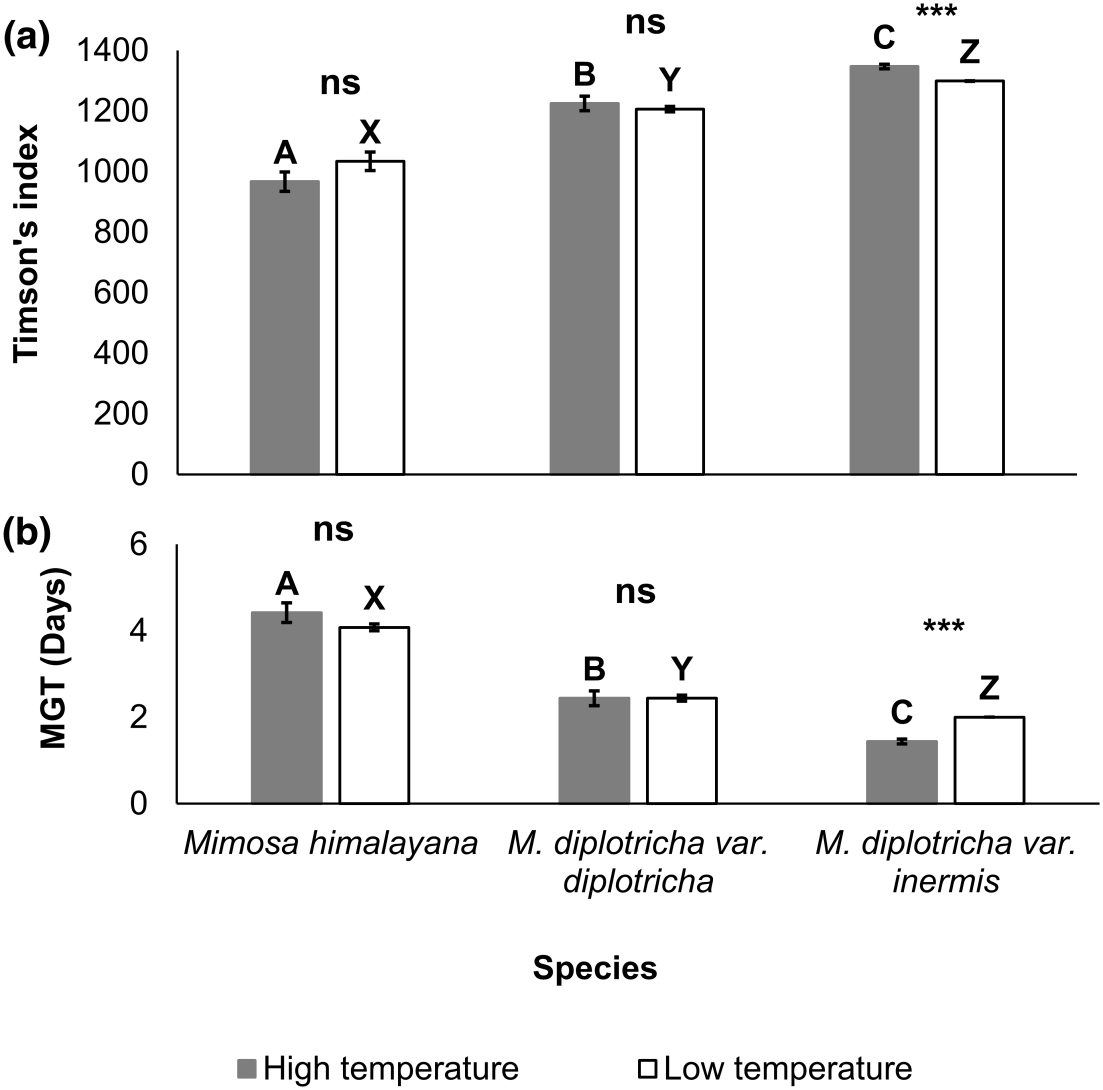
Variation of (a) Timson's index and (b) mean germination time (MGT) across species and between temperature treatments. Different alphabets (A–C and X–Z) above bars indicate significant differences among taxa at high and low temperatures, respectively (ANOVA); *** represent significant difference (*p* < .001, independent sample *t*‐test) and ns represent no difference between high and low temperature within each taxon.

Timson's index (TI) was the highest in *M. diplotricha* var. *inermis* followed by *M. diplotricha* var. *diplotricha* and *M. himalayana* at both high and low temperatures (Figure 2a). The MGT was the longest in *M. himalayana* followed by *M. diplotricha* var. *diplotricha* and *M. diplotricha* var. *inermis*, indicating that the invasive taxa reached to a maximum germination rate sooner than native taxon (Figure 2b). Both TI and MGT were independent of the exposed temperature regimes in *M. himalayana* and *M. diplotricha* var. *diplotricha* but they differed between high and low temperatures in *M. diplotricha* var. *inermis*.

### 3.3 Effect of water stress

**FIGURE 3:**
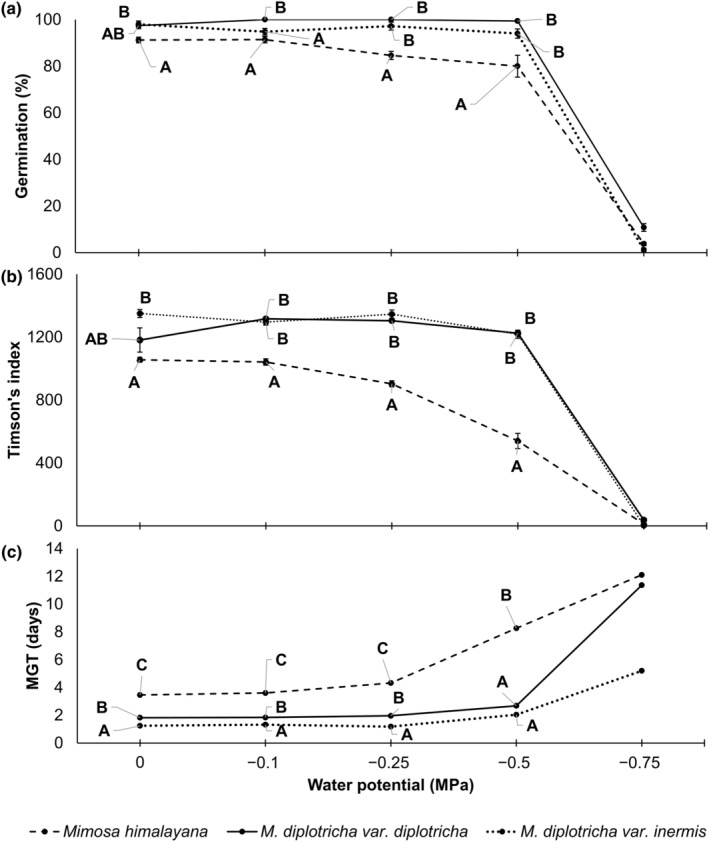
Effect of water stress on (a) germination percent (GP) (b) Timson's index (TI), and (c) mean germination time (MGT) at 25/15°C and a 12‐h photoperiod. Different alphabets A–C represent significant difference among species at each water potential (ANOVA). The ANOVA method was not done for the values at −0.75 MPa because of many zero values.

A decline in water potential (up to −0.5 MPa) resulted into a significant decrease in the GP and TI of native *Mimosa himalayana* but not of invasive *M. diplotricha* var. *diplotricha* and *M. diplotricha* var. *inermis* (Figure 3, Table A2). In particular, water stress up to −0.5 MPa water potential in *M. diplotricha* did not have effect on GP and TI but the effect was significant at −0.75 MPa. The MGT increased with a decline of water potential in all taxa but the increase was more pronounced in native than in the invasive taxa at −0.50 MPa water potential. Only few seeds of all taxa germinated at −0.75 MPa and no seeds germinated at −1 MPa.

The distinction in GP between native and invasive taxa was not clear at control and −0.10 MPa water potential but the GP was significantly lower in native taxon at −0.25 and −0.50 MPa (Figure 3a). For TI, the native–invasive difference was significant at −0.10, −0.25 and −0.50 MPa but not at control (Figure 3b). The MGT was significantly longer in native *M. himalayana* than in invasive taxa at all water potential treatments including control (Figure 3c). The two‐way ANOVA also revealed that the GP, TI, and MGT varied significantly with species, water potential as well as their interactions. The effects of species and water potential alone were higher compared with their combined effects on the MGT (Table 5).

**TABLE 5:** Results of two‐way ANOVA showing the effect of different variables on germination percentage (GP), Timson's index (TI), mean germination time (MGT), and emergence percentage.

Test parameters	Variables	Df	*f*	Sig.
Germination percentage	Taxa	2	63	<0.001
	Water Potential	4	533	<0.001
	Taxa × Water Potential	8	6	<0.001
Timson's index (TI)	Taxa	2	213	<0.001
	Water Potential	4	1000	<0.001
	Taxa × Water Potential	8	29	<0.001
Mean germination time (MGT)	Taxa	2	30	<0.001
	Water Potential	4	41	<0.001
	Taxa × Water Potential	8	3	<0.01
Seedling emergence percentage	Taxa	2	1	>0.05
	Depth	2	94	<0.001
	Taxa × Depth	4	1	>0.05

### 3.4 Effect of soil depths on seedling emergence

**FIGURE 4:**
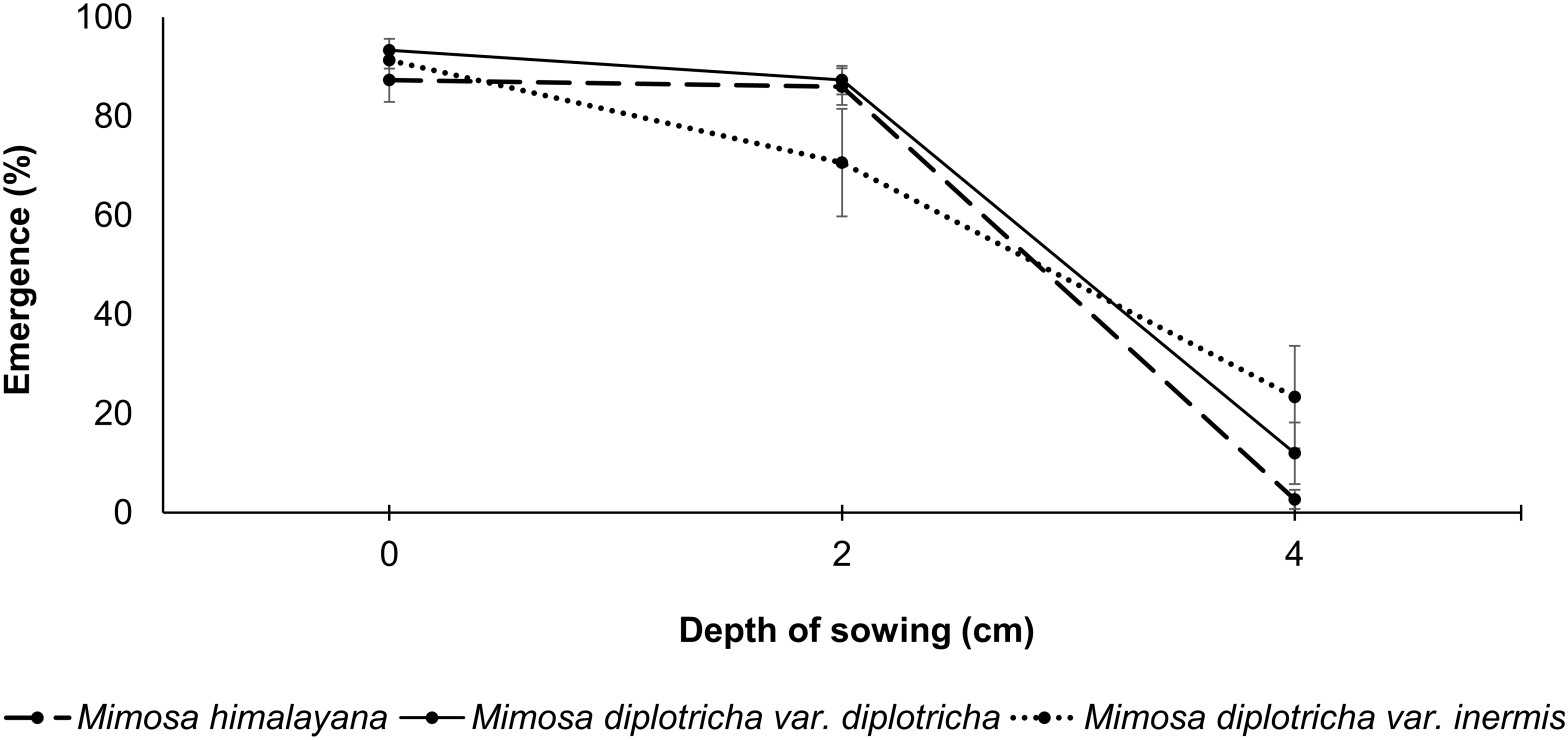
Effect of depth on seedling emergence. There was no difference in seedling emergence among taxa (*p* > .05, ANOVA).

**FIGURE A7:**
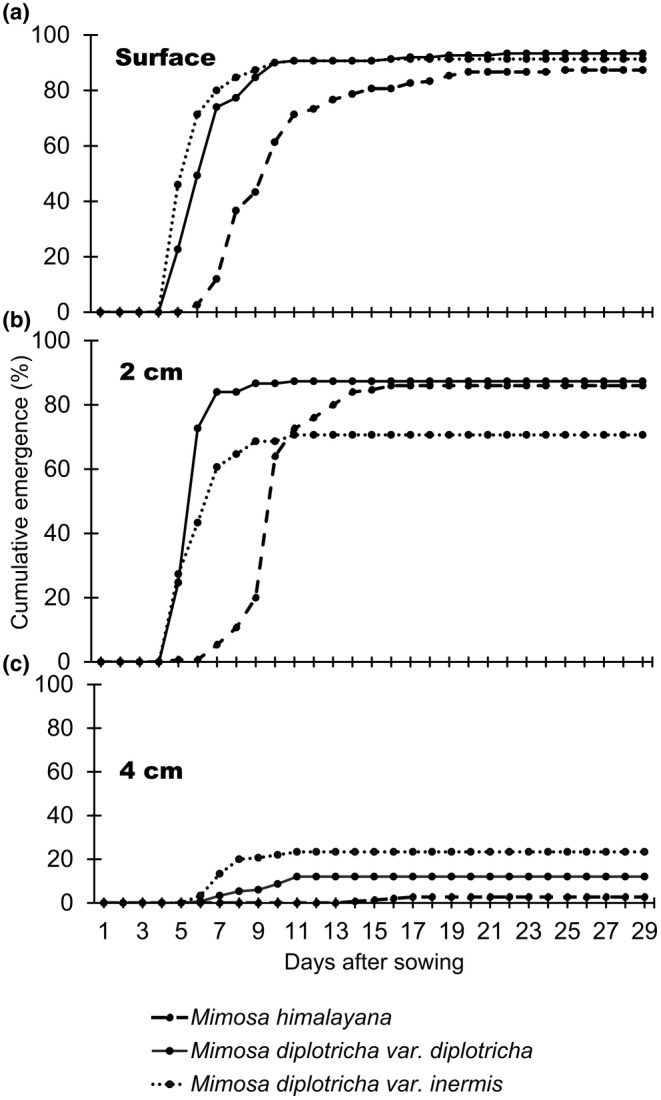
Cumulative seedling emergence percentage of seeds sowed at different depths: (a) surface, (b) 2 cm below soil surface, and (c) 4 cm below the soil surface. For each taxon and depth, 150 seeds were sown. Seeds sown below 4 cm did not emerge.

Seeds of all taxa germinated when sown up to 4 cm below soil surface; seedlings from the seeds sown at 6, 8, and 10 cm depth did not emerge out. At the termination of the experiment, when the seeds were checked in the pots, we did not find any intact seeds but we found decayed radicles and seed coats. Seedling emergence did not vary among taxa at each depth (Figure 4). Two‐way ANOVA revealed that seed sowing depth had impacts on seedling emergence, but it was not affected by taxon identity and its interaction with sowing depth. There was no significant difference in seedling emergence percent between surface and 2 cm depth of sowing in each taxa, but it was significantly lower when seeds were sowed at 4 cm depth in all three taxa (Table A3). Variation of cumulative emergence percentage showed that seedlings of invasive taxa emerged earlier and at faster rate than native (Figure A7).

### 3.5 Biomass allocation

**FIGURE 5:**
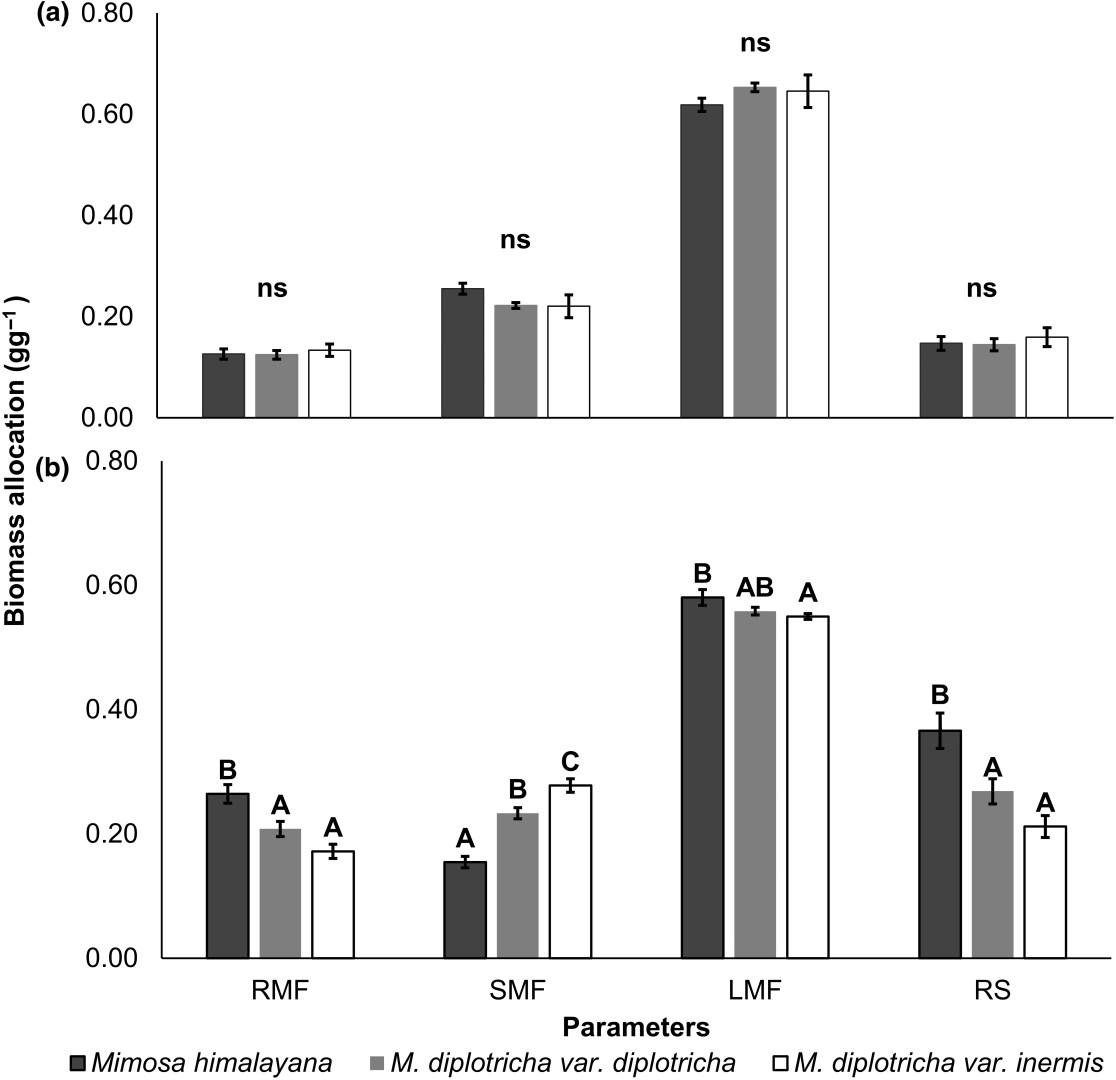
Root mass fraction (RMF), stem mass fraction (SMF), leaf mass fraction (LMF), and root shoot ratio (RS) among taxa at (a) 48 DAS and (b) 90 DAS. Different alphabets above bars represent significant differences among taxa (*p* < .05, ANOVA). ns, not significant.

There was no significant difference in the RMF, SMF, LMF, and root shoot ratio (RS) of seedling among study taxa in the first harvest (48 days after sowing, DAS) (Figure 5a). However, these attributes varied across taxa in seedlings harvested at 90 DAS (Figure 5b). The RMF and LMF were higher in native *Mimosa himalayana* than in invasive congener. But, the SMF was the highest in the invasive *M. diplotricha* var. *inermis*. The RS was higher in native species than in both varieties of the invasive species. Two varieties of *M. diplotricha* also differed in SMF but not in RMF, LMF, and RS (Figure 5b).

Biomass allocation patterns changed with seedling harvest date in all three taxa (Table A4). The RMF and RS increased but the LMF declined in the successive harvests in all taxa. However, variation of the SMF did not show any consistent pattern and was taxon‐specific.

### 3.6 Seedling growth and vigor

**FIGURE 6:**
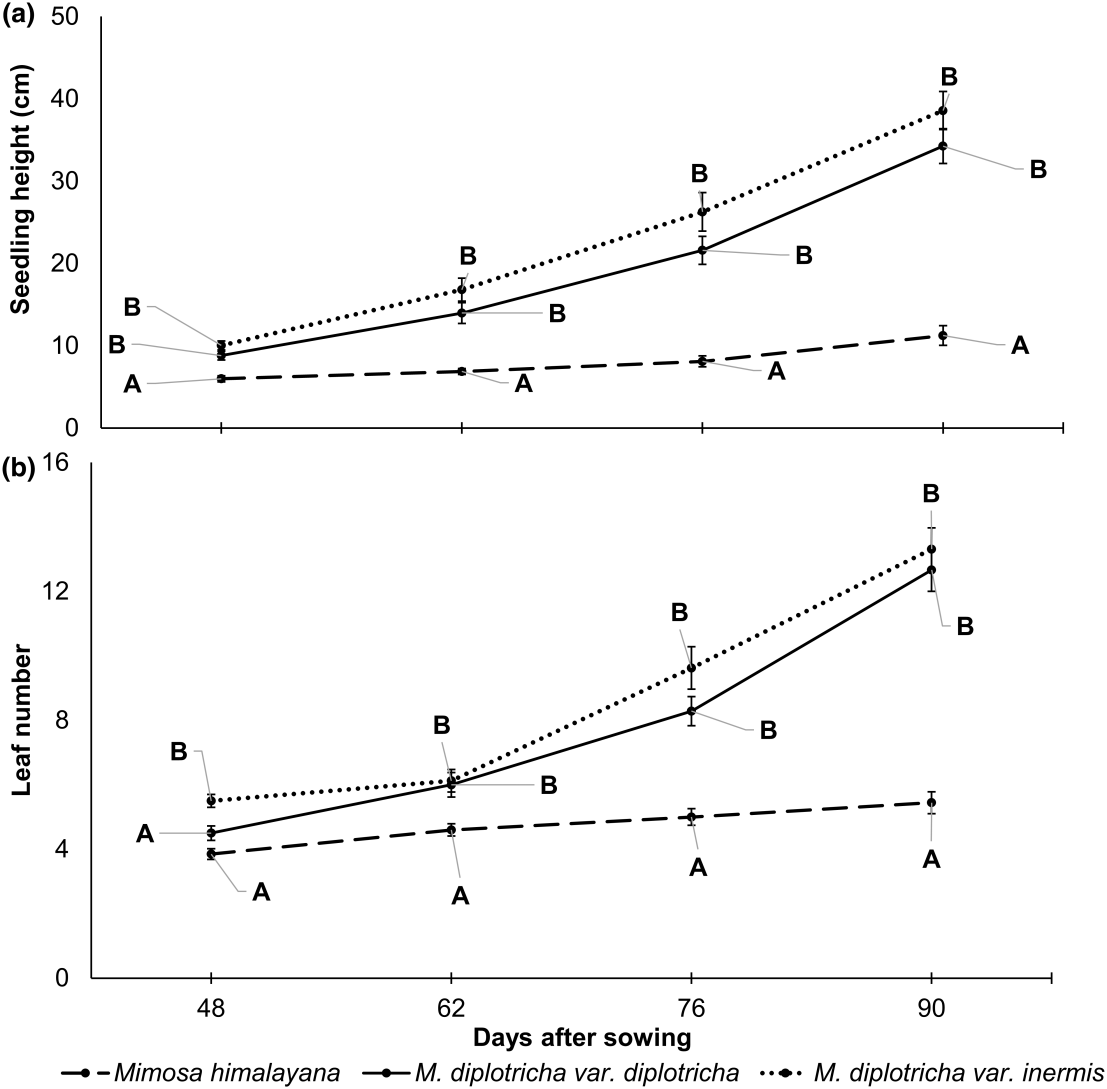
Change in (a) seedling height and (b) leaf number of seedling in successive harvests. Different alphabets A–C above the line represents significant difference among taxa at each harvest (*p* < .05, ANOVA).

RGR of two varieties of the invasive species were nearly equal but it was nearly two times as high as it was for the native species (Table 6). As expected, seedling height and the number of leaves in each seedling of all taxa increased with harvest dates, but the increase was more pronounced in invasive species than in native. For example, seedling height at 90 DAS was 45% higher than at 48 DAS in native *M. himalayana* but it was 277% and 290% in invasive *M. diplotricha* var. *diplotricha* and *M. diplotricha* var. *inermis*, respectively (Figure 6, Table A5). Similarly, the number of leaves at 90 DAS was 25% higher than that at 48 DAS in native species but it was 160% in *M. diplotricha* var. *diplotricha* and 117% in *M. diplotricha* var. *inermis*. Seedling height and the number of leaves did not vary between two varieties of invasive species at different harvest date (except for leaf number at 48 DAS), but it was always higher in invasive species than in native species (Figure 6). Native–invasive differences increased with DAS of the seedlings. For example, seedling height of invasive species (mean of two varieties) was 58% higher than that of native species at 48 DAS but it was 332% at 90 DAS. Similarly, the number of leaves of invasive species was 38% higher than that of native species at 48 DAS but it was 160% at 90 DAS.

**TABLE 6:** Relative growth rate of *Mimosa* species.

Taxa	Relative growth rate (mg/g/day)
*Mimosa himalayana*	40
*Mimosa diplotricha* var. *diplotricha*	74
*Mimosa diplotricha* var. *inermis*	70

## 4 DISCUSSION

Invasion scientists are in search of organisms' traits which are explicitly associated with high invasiveness and thus can be used for the identification of potentially invasive species during risk assessments. By comparing seed germination and seedling growth traits of congeneric native (with no known introduced population) and invasive *Mimosa* species, we showed that the invasive species had higher germination rate (Timson's index), shorter MGT, higher seedling RGR, and greater seedling height than the native species. These results suggest that the invasive *M. diplotricha* show some of the characteristic features of the “ideal weed” (sensu Baker, 1974) such as the capacity to germinate under wide environmental conditions (e.g., photoperiod and continuous dark, higher water stress condition) and adaptation to long distance dispersal (as a result of physical dormancy). These results have direct implications in the risk assessment of invasive species because the invasive species predictability can be improved when these traits are taken into account during the risk assessment process.

### 4.1 Seed attributes

Seed attributes such as mass and size are often associated with plant's life history strategies that promote invasiveness (Grotkopp et al., 2002; Maranon & Grubb, 1993). The results of the present study showed that seeds of invasive species were smaller than that of a native congener. Species with small seeds are more likely to be invasive as small seed mass appears to be linked with greater seed production and higher RGR which are critical for the successful establishment of invasive species (Aarssen & Jordan, 2001; Grotkopp et al., 2002; Maranon & Grubb, 1993; Moodley et al., 2013; Reichard, 2003; Wright & Westoby, 1999). In a comparison between invasive and native species in Indonesia, Rindyastuti et al. (2021) found that the invasive species has lower seed mass compared with the native species. However, a global level comparison could not find a significant difference in seed mass between native and invasive species though the former tended to have smaller seeds (Mason et al., 2008). Gioria et al. (2021) also reported that seed mass is weakly associated with invasiveness and the relationship between seed mass and invasiveness can vary depending on various other factors like the spatial scale of the study as well as the plant's life form. Despite a large difference in seed mass between native and invasive species in the present study, disagreements among previous studies suggest that this seed trait cannot be a definitive predictor of invasiveness.

### 4.2 Environmental effects on seed germination

It is generally anticipated that high seed germination rate confers high invasiveness (Baker, 1974; Goergen & Daehler, 2001; Schlaepfer et al., 2010). In the present study, the invasive species tended to have higher GP than native species but the pattern was inconsistent because the native–invasive distinction disappeared in some treatments (e.g., high temperature under continuous dark condition). However, in a comparison between native (12 spp.) and co‐occurring naturalized species (12 spp.) of coastal sage scrub (California), Wainwright and Cleland (2013) showed that the naturalized species had higher seed GP than native species. Higher seed GP has been also reported in invasive *Ardisia elliptica* than sympatric native *A. obovata* (Munoz & Ackerman, 2011). In the present study, the invasive species also had significantly higher germination rate (Timson's index) and shorter time to maximum germination than native species at both low and high temperatures. Such early germination of invasive species may have an advantage over the coexisting native species in survival and growth in competitive environments due to space preemption and greater access to resources, which could increase the likelihood of successful establishment (Byun, 2023; Guido et al., 2017).

The results of the present study revealed that the seed germination of both native as well as invasive taxa were independent of light. Such seeds can therefore germinate whether it is buried into the soil or exposed as long as moisture and temperature conditions are favorable (Chauhan & Johnson, 2008). Such light indifference in germination has been reported as a common phenomenon of most members of Fabaceae (Baskin et al., 1998; Baskin & Baskin, 2014; Chauhan & Johnson, 2008, 2009; Silveira & Fernandes, 2006) and this can be attributed to a relatively high seed mass of both native and invasive taxa in the present study (Milberg et al., 2000; Pearson et al., 2002).

Water stress often has differential effects on germination of native and invasive species (Cervera & Parra‐Tabla, 2009; Perez‐Fernandez et al., 2000). In the present study, the differences in GP, Timson's index and MGT between native and invasive were small but statistically significant. Higher GP and rate of both varieties of invasive species than that of the native one, with larger differences at lower water potential, suggests that invasive species have higher tolerance to water stress than native ones. This ability of invasive species to germinate better than native under water stress conditions could enable them to gain advantage as a weed because of earlier seedling emergence (during pre‐monsoon in the study area) under water stress conditions (Byun, 2023; Chauhan & Johnson, 2008).

Seedling emergence declined with increasing depth of seed sowing, independent of species—a pattern reported by several previous studies (e.g., Chauhan & Johnson, 2008, 2009; Hao et al., 2017). Lack of germination of the seeds sown below 4 cm could be attributed to the exhaustion of seedling reserves before emergence (Tamado et al., 2002). However, our results differed from Chauhan and Johnson (2008) who reported seedling emergence of *Mimosa diplotricha* from seeds sown up to 8 cm depth. This difference might be to the result of differences in substrate composition used for seedling emergence experiments. Recovery of seed coat and radicle fragments of the deep sown seeds might be the result of seed scarification prior to the emergence experiment, as scarification might have initiated the germination process in the deeply buried seeds, but the seedlings failed to emerge (Chauhan & Johnson, 2008, 2009).

### 4.3 Biomass allocation

Native–invasive distinction among study taxa was blurred in LMF but clear in RMF, SMF, and RS. Lower RMF and RS as well as higher SMF of invasive *Mimosa diplotricha* can be attributed to a heliophytic nature of this species (Uyi, 2020), which require greater biomass allocation to stem for making them taller and more competitive (Delagrange et al., 2004). As both varieties of the invasive *M. diplotricha* are creeping plants, they adapt for additional height growth by elongating stems through additional resources allocation (Poorter et al., 2012). Both the varieties of *M. diplotricha* showed similar growth performance. A similar growth pattern has been also reported by Wang et al. (2019) when these two varieties were grown under ambient environmental conditions. Invasive plants often exhibit greater biomass allocation to shoot (lower RS) during early stages than native species, which may increase carbon assimilation efficiency and thereby reduce constraints to the establishment of invasive species in a community (Daehler, 2003; Grotkopp et al., 2002; Rejmánek & Richardson, 1996; Van Kleunen et al., 2010). Additionally, greater allocation to shoot as compared with root may confer high competitiveness over the slow‐growing native species in their habitats with long‐term ecological consequences.

### 4.4 Seedling growth vigor

Higher RGR and greater height of invasive *Mimosa diplotricha* than that of native *M. himalayana* suggests a close association between these traits and invasiveness. Higher RGR of invasive species have been also reported in studies comparing invasive species with co‐occurring (e.g., Kumar & Garkoti, 2021) or phylogenetically closely related (con‐familial) native species (e.g. Airi et al., 2023). A similar association between RGR and species invasiveness has also been suggested by Pyšek and Richardson (2007) but such an association has been questioned by Daehler (2003) based on a meta‐analysis involving 79 independent native–invasive comparisons. However, there are strong evidences on that greater plant height gives higher invasiveness among naturalized species (Divíšek et al., 2018). The ecological benefit of a high RGR enables invasive species to quickly occupy space, capture resources, and shorten the interval between vegetative growth and reproduction (Assad et al., 2021; Poorter, 1989). Most invasive species begin as small naturalized populations but at later stages show accelerated population growth facilitated by many key features that may play a role in their rapid spread, including high RGR (Grotkopp et al., 2002) and seed germination success (Van Kleunen et al., 2010). Invasive species with high seedling RGR may have an advantage in survival and growth in competitive environments (i.e., outcompete native species) due to space preemption and greater access to resources, which may have a substantial impact on coexisting native species (Guido et al., 2017).

## 5 CONCLUSIONS

Comparison of seed germination patterns and seedling growth traits of native *Mimosa himalayana* with co‐occurring two varieties of congeneric invasive species (*M. diplotricha* var. *diplotricha* and *M. diplotricha* var. *inermis*) revealed that some traits such as the higher germination rate (Timson's index), shorter MGT, higher seedling relative growth rate, and greater seedling height are associated with the invasive species. The results suggest that invasive species predictability of the risk assessment tools can be improved when these traits are taken into account during the risk assessment process. Future laboratory experiments on interactions between seedlings of native and invasive species under different environmental conditions can provide better insights on their relative performance when they co‐occur in natural habitats. Similarly, additional studies involving multiple native–invasive pairs representing different genera and families from data poor regions such as the South Asia may help to test the generality of the usefulness of germination and seedling growth traits in risk assessment process.

## AUTHOR CONTRIBUTIONS

**Nisha Kharel:** Data curation (lead); formal analysis (lead); investigation (lead); visualization (lead); writing – original draft (lead). **Anuj Dangol:** Data curation (equal); investigation (equal); writing – review and editing (supporting). **Ashmita Shrestha:** Data curation (equal); investigation (equal); writing – review and editing (supporting). **Hemanti Airi:** Data curation (equal); investigation (equal); writing – review and editing (supporting). **Anjana Devkota:** Conceptualization (equal); funding acquisition (equal); investigation (equal); methodology (equal); resources (equal); writing – review and editing (equal). **Lal Bahadur Thapa:** Conceptualization (equal); funding acquisition (equal); methodology (equal); resources (equal); writing – review and editing (equal). **Bharat Babu Shrestha:** Conceptualization (lead); data curation (supporting); formal analysis (supporting); funding acquisition (lead); investigation (equal); methodology (lead); project administration (lead); resources (lead); supervision (lead); validation (lead); visualization (equal); writing – original draft (supporting); writing – review and editing (lead).

## FUNDING INFORMATION

This research was financially supported by The World Academy of Sciences (TWAS) (grant no. 20‐269 RG/BIO/AS_G–FR3240314162).

## CONFLICT OF INTEREST STATEMENT

The authors have no relevant financial or non‐financial interests to disclose.

## Data Availability

All data used in this manuscript has been included in this manuscript including the Appendix at the end of this manuscript.

## APPENDIX A

**TABLE A1:** Number of seedlings of *Mimosa* species at different harvest time.

Days after sowing (DAS)	*Mimosa himalayana*	*Mimosa diplotricha* var. *diplotricha*	*Mimosa diplotricha* var. *inermis*
48 DAS	20	20	20
62 DAS	17	17	19
76 DAS	14	19	18
90 DAS	12	20	18

**TABLE A2:** Effect of water potentials on germination parameters (mean ± SE).

Parameters	Water potential (MPa)	*Mimosa himalayana*	*Mimosa diplotricha* var. *diplotricha*	*Mimosa diplotricha* var. *inermis*
Germination percent	0	91 ± 1^b^	97 ± 1^p^	98 ± 1^x^
	−0.1	91 ± 1^b^	100 ± 0^p^	95 ± 1^x^
	−0.25	85 ± 2^ab^	100 ± 0^p^	97 ± 2^x^
	−0.5	80 ± 5^a^	99 ± 0.6^p^	94 ± 2^x^
	−0.75	4 ± 1	11 ± 2	1 ± 1
Timson's index	0	1056 ± 15^c^	1182 ± 77^p^	1350 ± 25^y^
	−0.1	1042 ± 20^c^	1317 ± 2^p^	1295 ± 19^xy^
	−0.25	903 ± 19^b^	1305 ± 3^p^	1346 ± 27^y^
	−0.5	540 ± 48^a^	1225 ± 13^p^	1219 ± 26^x^
	−0.75	11 ± 1	37 ± 7	2.6 ± 2
Mean germination time	0	3.5 ± 0.1^a^	1.8 ± 0.04^p^	1.2 ± 0.1^x^
	−0.1	3.6 ± 0.1^a^	1.8 ± 0.02^p^	1.3 ± 0.2^x^
	−0.25	4.3 ± 0.2^a^	2.0 ± 0.02^p^	1.2 ± 0.1^x^
	−0.5	8.3 ± 0.4^b^	2.7 ± 0.1^q^	2.0 ± 0.02^y^
	−0.75	12.1 ± 0.1	11.4 ± 0.6	5.2 ± 3.2

*Note*: Different alphabets a–c, p–q and x–y represent significant difference across water potential levels for *Mimosa himalayana*, *Mimosa diplotricha* var. *diplotricha* and *Mimosa diplotricha* var. *inermis*, respectively (*p* ≤ .05, ANOVA).

**TABLE A3:** Effect of seed sowing depth on seedling emergence (mean ± SE).

Depth of sowing	*Mimosa himalayana*	*Mimosa diplotricha* var. *diplotricha*	*Mimosa diplotricha* var. *inermis*
Surface	87 ± 4^b^	93 ± 2^q^	91 ± 2^y^
2 cm	86 ± 4^b^	87 ± 3^q^	71 ± 11^y^
4 cm	3 ± 2^a^	12 ± 6^p^	23 ± 10^x^

*Note*: Different alphabets: a–b, p–q, and x–y represent significant difference among seed sowing depth for *Mimosa himalayana*, *Mimosa diplotricha* var. *diplotricha* and *Mimosa diplotricha* var. *inermis*, respectively (*p* ≤ .05, ANOVA).

**TABLE A4:** Comparison of root mass fraction (RMF), leaf mass fraction (LMF), stem mass fraction (SMF), and root shoot ratio (RS) at different harvests within species (DAS, days after sowing) (Mean ± SE).

Taxa	Parameters	48 DAS	62 DAS	74 DAS	90 DAS
*Mimosa himalayana*	RMF	0.13 ± 0.01^a^	0.14 ± 0.01^a^	0.29 ± 0.03^b^	0.26 ± 0.02^b^
	SMF	0.26 ± 0.01^b^	0.24 ± 0.01^b^	0.17 ± 0.01^a^	0.15 ± 0.01^a^
	LMF	0.62 ± 0.01^b^	0.62 ± 0.01^b^	0.54 ± 0.04^a^	0.58 ± 0.01^ab^
	RS	0.15 ± 0.01^a^	0.16 ± 0.01^a^	0.44 ± 0.07^b^	0.37 ± 0.03^b^
*Mimosa diplotricha* var. *diplotricha*	RMF	0.12 ± 0.01^p^	0.23 ± 0.03^q^	0.21 ± 0.02^q^	0.21 ± 0.01^q^
	SMF	0.22 ± 0.01^pq^	0.21 ± 0.01^p^	0.25 ± 0.01^q^	0.23 ± 0.01^pq^
	LMF	0.65 ± 0.01^q^	0.56 ± 0.02^p^	0.54 ± 0.01^p^	0.56 ± 0.01^p^
	RS	0.14 ± 0.01^p^	0.33 ± 0.08^q^	0.27 ± 0.03^q^	0.27 ± 0.02^q^
*Mimosa diplotricha* var. *inermis*	RMF	0.13 ± 0.01^x^	0.17 ± 0.01^y^	0.14 ± 0.01^xy^	0.17 ± 0.01^xy^
	SMF	0.22 ± 0.02^x^	0.24 ± 0.01^xy^	0.28 ± 0.01^y^	0.28 ± 0.01^y^
	LMF	0.65 ± 0.03^y^	0.59 ± 0.01^xy^	0.58 ± 0.01^x^	0.55 ± 0.01^x^
	RS	0.16 ± 0.02^x^	0.21 ± 0.01^x^	0.17 ± 0.02^x^	0.21 ± 0.02^x^

*Note*: Different alphabets a–b, p–q and x–y represent significant difference within *Mimosa himalayana*, *Mimosa diplotricha* var. *diplotricha* and *Mimosa diplotricha* var. *inermis*, respectively, at different harvests (*p* ≤ .05, ANOVA).

**TABLE A5:** Variation of leaf number and seedling height (mean ± SE) of the study taxa with different harvests time (DAS, days after sowing)*.*

Attributes	Taxa	48 DAS	62 DAS	76 DAS	90 DAS
Seedling height (cm)	*Mimosa himalayana*	6 ± 0.4^a^	7 ± 0.4^a^	8 ± 0.7^a^	11 ± 1.2^b^
	*Mimosa diplotricha* var. *diplotricha*	9 ± 0.5^p^	14 ± 1.3^q^	22 ± 1.7^r^	34 ± 2.1^s^
	*Mimosa diplotricha* var. *inermis*	10 ± 0.5^w^	17 ± 1.4^x^	26 ± 2.3^y^	39 ± 2.3^z^
Leaf number	*Mimosa himalayana*	4 ± 0.2^a^	5 ± 0.2^ab^	5 ± 0.3^b^	5 ± 0.3^b^
	*Mimosa diplotricha* var. *diplotricha*	5 ± 0.2^p^	6 ± 0.4^p^	8 ± 0.5^q^	13 ± 0.7^r^
	*Mimosa diplotricha* var. *inermis*	6 ± 0.2^w^	6 ± 0.4^w^	10 ± 0.7^x^	13 ± 0.7^y^

*Note*: Different alphabets in superscript in each row represent a significant difference (*p* ≤ .05, ANOVA).
